# Exploring the Pathogenic Potential of *Vibrio vulnificus* Isolated from Seafood Harvested along the Mangaluru Coast, India

**DOI:** 10.3390/microorganisms8070999

**Published:** 2020-07-04

**Authors:** Caroline D’Souza, Kattapuni Suresh Prithvisagar, Vijay Kumar Deekshit, Indrani Karunasagar, Iddya Karunasagar, Ballamoole Krishna Kumar

**Affiliations:** 1 Division of Infectious Diseases, Nitte University Centre for Science Education and Research, Nitte (Deemed to be University), Deralakatte, Mangalore 575018, India; carolinedsouza90@gmail.com (C.D.); sagarputtur24@gmail.com (K.S.P.); deekshit1486@nitte.edu.in (V.K.D.); indrani.karunasagar@nitte.edu.in (I.K.); 2 Nitte (Deemed to be University), University Enclave, Medical Sciences Complex, Deralakatte, Mangaluru 575018, India; iddya.karunasagar@nitte.edu.in

**Keywords:** foodborne pathogen, *Vibrio vulnificus*, septicemia, C/E genotype, serum susceptibility, virulence gene expression, cytotoxicity

## Abstract

It has been observed that not all strains of *Vibrio vulnificus* are virulent. Determining the virulence of strains that are frequently present in seafood is of significance for ensuring seafood safety. This study is an attempt to predict the virulence of seafood-borne *V. vulnificus* isolated along the Mangaluru Coast, India. The isolates tested possessed a *vcgC* gene sequence with high similarity to that in the clinical strain. Transcriptional analysis of core virulence genes in seafood isolate E4010 showed the phenomenon of contact-mediated expression of *rtxA1* which correlated well with the actin disintegration and cytotoxicity. These results suggest that the seafood isolates tested in this study possess a functional RtxA1 which could help in initiating the infection. However, other putative virulence genes such as *vvpE* encoding an extracellular protease, *vvhA* encoding hemolysin, *flp* encoding tad pilin and *ompU* encoding fibronectin-binding protein were also constitutively expressed. Virulence-associated attributes such as cytotoxicity and adherence matched the response of the clinical strain (*p* > 0.05). On the other hand, the environmental strains showed higher serum sensitivity compared with the clinical strain. These findings show that the part of virulence attributes required for the disease process might be intact in these isolates.

## 1. Introduction

*V. vulnificus* is a Gram-negative opportunistic human pathogen that is widely disseminated in marine environments around the world [1,2]. This organism is capable of causing life-threatening septicaemia, wound infections and acute gastroenteritis, especially in susceptible or immune-compromised individuals, and has a mortality rate of over 50% [3]. The cases of human infections due to *V. vulnificus* occur either by the ingestion of molluscan seafood or by direct entry through open wound exposure to seawater [4]. The bacterium is often found in molluscan seafood with the highest occurrence rate in oysters. [5,6,7,8]. *V. vulnificus* infections have been reported around the world, with cases frequently reported from the United States, Europe and Korea [1]. However, *V. vulnificus* infections are often unrecognized and under-reported in India [9,10]. The organism is highly lethal and accounts for over 95% of seafood-related deaths in the United States [3]. The mortality rate associated with primary septicaemia is almost similar to risk group-3 (RG-3) and risk group-4 (RG-4) pathogens [2]. These categories include the most serious and lethal pathogens which are dealt with stringent lab precautions and maximum containment [11].

Considering the pathogenic potential of this organism, several virulence typing schemes have been developed. *V. vulnificus* subtyping based on polymorphism of 16S rRNA, Virulence correlated gene (*vcg*) and Capsular polysaccharide (CPS) are the commonest approaches. The 16S rRNA type A variation significantly correlates with isolates having environmental origin whereas type B correlates with clinical isolates [12]. DNA sequence variation in *vcg* subdivides the population into clinical (*vcgC* genotype) and environmental (*vcgE* genotype) origin [13]. Two distinct alleles associated with CPS operon corresponds to two genotypes. The HP1 allele polymorphism is observed in clinical and HP2 is commonly observed in nonclinical types [14].

There are many attributes to distinguish virulent and nonvirulent strains, but all have limitations. Consistency and exclusivity of these markers in predicting the virulence potential still remains in question [2,15]. Environmental isolates tested for mouse virulence demonstrated no discernible difference with the clinical strain [16,17,18,19]. All clinical and seafood (environmental) isolates with haemolytic activity possessed the *vvhA* gene encoding hemolysin/cytolysin [17,19,20]. The gene encoding hemolysin/cytolysin is highly conserved and hence used as a marker for the species identification. MARTX (Multifunctional Autoprocessing Repeat-in Toxin) encoded by *rtxA1* is a key virulence factor in bacterial pathogenesis, responsible for cytopathic and cytotoxic effects [21]. However, variation in the presence of *rtxA1*, *vvpE* (encoding metalloprotease), *viuB* (encoding vulnibactin) and serum susceptibility among environmental isolates in comparison to clinical isolates was reported earlier [20,22,23]. The siderophore vulnibactin, encoded by *viuB*, is the factor contributing to iron acquisition by *V. vulnificus*. Iron availability seems to be the critical factor for bacterial survival in serum [24]. Previous studies revealed that a lack of the *viuB* gene in most environmental isolates contributed to decreased virulence potential [20,23,25]. Hence, this gene was earlier used as one of the markers to differentiate pathogenic and nonpathogenic strains [26]. Later studies showed that this gene was present in 80–100% of environmental isolates; hence, it no longer a marker for virulence [27,28]. 

The pilus of *V. vulnificus* was first identified in 1989 with most clinical isolates possessing them and environmental isolates lacking them [29]. Expression analysis of the pilin genes during infection indicated higher expression in virulent isolates than in the less virulent isolates [30]. Mutational analysis revealed the role of both *pilA* (encodes for pilin structural peptidase) and *pilD* (encodes for prepilin peptidase) in adherence [31,32]. The association of capsular polysaccharides and virulence has also been documented [29]. The role of capsular polysaccharides in the survival of this organism in serum has been surmised; isolates presenting as opaque colonies are more resistant to serum than translucent isolates with a difference in colony characteristics between seafood and clinical isolates. Clinical isolates have shown greater resistance to serum complement proteins than environmental isolates and the C genotype has a consistent survival advantage when exposed to serum [22,33,34]. 

Although the presence of *V. vulnificus* in seafood harvested from India has been recorded, there is a paucity of reports of infection. To date, only five reports of *V. vulnificus* infections have been documented in India [9,10,35,36,37]. The incidence of *V. vulnificus* in marine fish samples was found to be 16·6% [38]. The overall incidence of *V. vulnificus* was low in seafood, whereas oysters proved to be a rich source of *V. vulnificus* with occurrence rates of 81–85% [5,6,39]. While the ecology of the organism has received considerable research attention, fundamental questions regarding the pathogenic ability of the organism remain unanswered. To date, there are no studies on the characterization of seafood isolates from India in terms of their virulence properties. Hence, identifying the virulence and pathogenic potential of seafood-associated isolates is important from a public health point of view to ensure safety of seafood to consumers. In this study, biochemical assays, genotyping, ability to adhere to HeLa cells, the damage they cause to the cell structures, and expression level of some of the key virulence genes during infection of HeLa cells was performed. These experiments intended to examine the differences, if any, between clinical and seafood isolates, to provide insights into the pathogenic potential and allow for a risk assessment of *V. vulnificus* associated with seafood.

## 2. Materials and Methods 

### 2.1. Isolation and Identification of V. vulnificus

During the study period, 70 seafood samples were collected from fish markets and retailers along the Mangaluru coast (coast length of 50 km) from 03-01-2017 to 10-04-2018. Information on sampling location, the type of seafood samples analyzed and their positivity are detailed in Tables S3 and S4. Isolation of *V. vulnificus* from the seafood samples was as per the U.S. Food and Drug Administration (FDA’s) Bacteriological Analytical Manual (BAM) [40], with brief modification. This study protocols were approved by the Central Ethics Committee of the Nitte (Deemed to be University), Mangaluru (NU/CEC/2017-18/0122). All experiments were conducted in accordance with Institutional Biosafety Guidelines with consent from the Institutional Biosafety Committee, Nitte (Deemed to be University), Mangaluru. The samples were homogenized and 25 g of the homogenate was added to 225 mL of alkaline peptone water containing 20 U/mL of polymyxin (APW-P) and incubated at 37 °C. The pre-enrichment broth was drawn at 0, 2, 4, 6 and 18 h and a loopful was streaked on Cellobiose Colistin (CC) agar and incubated at 37 °C for 18 h. Flat, yellow colonies with the size ranging from 2 to 3 mm were streaked onto Luria Bertani (LB) agar for purification and a single colony was picked up and stored for further studies.

### 2.2. Biochemical Characterization

Biochemical and physiological tests performed on purified isolates included a test for indole, salt tolerance, citrate utilization, amino acid decarboxylase, lactose and mannitol utilization, oxidation/fermentation, catalase and oxidase [40]. Hemolytic activity was tested on LB agar supplemented with human erythrocytes (5% *v/v*) and lecithinase activity tested on egg yolk agar (HiMedia Laboratories Pvt. Ltd., Mumbai, India), wherein an opaque zone around the colony was considered positive. Chromo Azurol S (CAS) assay was done for the siderophore production according to Schwyn and Neilands (1987) [41]. Caseinase production was studied on skim milk agar (HiMedia Laboratories Pvt. Ltd., Mumbai, India) with a zone of clearance around the colony recorded as positive. Starch hydrolysis was performed on starch agar (HiMedia Laboratories Pvt. Ltd., Mumbai, India). After incubation around 24–48 h, the culture plate was flooded with gram’s iodine. A colorless zone of clearance surrounding the colony was considered positive. 

### 2.3. Genotyping and Detection of Virulence Genes of V. vulnificus

Biochemically suspect *V. vulnificus* isolates were grown in 5 mL of LB broth at 37 °C overnight with shaking. DNA was extracted by cetyl-trimethyl ammonium bromide (CTAB)-proteinase K method [42]. Species-specific confirmation of *V. vulnificus* was done by PCR targeting the conserved region of the gene coding DNA gyrase subunit B or topoisomerase II (*gyrB*) [7]. *V. vulnificus* reference strain MTCC 1145 was used as the control. Isolates were screened for a panel of virulence-associated genes (*vvhA, rtxA1*, *viuB),* as previously described [26,43,44]. Genotyping based on polymorphisms in *vcg* was done by PCR according to the method described by Rosche and colleagues [13]. *V. vulnificus* genotypes associated with CPS alleles were identified using PCR according to Han et al. [25]. The PCR products for representative samples were sent for sequence confirmation (Eurofins, Bangalore, India). All the PCR assays were carried out in a 30 µl volume containing 1 × buffer (HiMedia Laboratories Pvt. Ltd., Mumbai, India), 50 µM each of the four deoxynucleotide triphosphates (dNTPs), 300 nM of each primer, and 1.0 U of *Taq* DNA polymerase (HiMedia Laboratories Pvt. Ltd., Mumbai, India). Two microliters of the genomic DNA (100–200 ng/uL) was used as a template. The reaction was carried out with the initial denaturation at 95 °C for 10 min followed by 35 cycles of denaturation at 95 °C for 30 s, respective annealing temperature for 30 s and elongation at 72 °C for 20 s. Details of the primers used and their respective annealing temperature are provided in Table S5. PCR products were electrophoresed in an agarose gel containing ethidium bromide (0.5 μg/mL) and images were captured using a gel documentation system (Bio-Rad, Hercules, CA, USA).

### 2.4. Phylogeny Tree Construction

Phylogeny was estimated in the Molecular Evolutionary Genetics Analysis (MEGA) program, Version 5.0 by the Neighbor-joining method (p-distance) using the sequence information obtained from the NCBI GenBank database along *vcg* alleles sequenced in this study (*V. vulnificus* E3716 and E4010).

### 2.5. Bacteria Strains Used for Virulence Assay

Based on the RAPD pattern obtained, four representative seafood isolates (E4010, E3715, E3716 and E3717) were selected for virulence assays including adherence, cytotoxicity, invasion and serum sensitivity. Analysis of structural damage of the HeLa cell membrane and estimation of virulence gene expression at the time of infection were carried out using environmental isolate E4010. For the comparison of virulence assays, clinical isolate K4633 was used, which was obtained from a patient with septicaemia (generously provided by Dr Jessica L Jones, US Food and Drug Administration). 

### 2.6. Serum Resistance 

Blood was collected from five healthy individuals, allowed to clot, and the serum separated by centrifugation at 1000× *g* for 10 min at 4 °C. Dilutions of serum were made in 1× PBS for final concentrations at 100, 50, 25, 12.5, and 6.25%. *V. vulnificus* strains were grown overnight in LB broth at 37 °C, washed once with 1 × PBS and diluted to achieve the final bacterial concentration of 10^6^ CFU/mL. The control mixture contained heat-inactivated (56 °C for 30 min) serum at different dilutions. The samples were incubated at 37 °C and 100 μL of the samples were plated onto LB agar every 30 min for 3 h. 

### 2.7. Adherence and Invasion Assays

HeLa cells were grown in Dulbecco’s Modified Eagle’s Medium (DMEM) with glucose (0.45%) (HiMedia Laboratories Pvt. Ltd., Mumbai, India) supplemented with 10% (*v/v*) fetal bovine serum (FBS) (PAN-Biotech, Aidenbach, Bavaria, Germany) in 12-well cell culture plates (Eppendorf, Hamburg, Germany) at 37 °C in 5% CO_2_ (Panasonic, Kadoma, Osaka, Japan). Cells were grown to 80–90% confluency (c. 0.4 × 10^6^ cells), washed with phosphate-buffered saline (PBS) thrice to remove floating cells and residual antibiotics. For infection studies, bacterial cells were grown in 50 mL LB broth with 1% NaCl until an optical density of approximately 0.60 (1 × 10^8^ CFU/mL). These cultures were used to infect HeLa cells at a multiplicity of infection (MOI) of 1:5. The plate was centrifuged at 300 × *g* for 5 min to ensure maximum adherence to the cell monolayer, which was then incubated at 37 °C for 1, 3 and 5 h. At each time point, HeLa cells were washed thrice with Dulbecco’s PBS (D-PBS) (HiMedia Laboratories Pvt. Ltd., Mumbai, India) to remove the nonadherent extracellular bacterial cells. The infected cells were detached and lysed using trypsin/EDTA (HiMedia Laboratories Pvt. Ltd., Mumbai, India) and were harvested to microcentrifuge tubes. To estimate the number of adhered bacteria, 100 µL of cell suspension was serially diluted in 1 × PBS followed by plating onto LB agar. The experiment was replicated and the percentage of adherence was calculated based on the number of recovered bacteria (CFU) by considering the CFU inoculated as 100. To estimate the cell invasive potential of the bacteria, gentamicin protection assay was performed. Bacterial cells at a MOI of 1:5 were added to each well and incubated for 30 min at 37 °C. This was followed by incubation cells that were washed with 1 × PBS to remove extracellular bacteria and treated DMEM containing gentamycin (100 μg/mL) for 30 min to kill any extracellular/adherent bacteria. The cells were re-incubated with DMEM at 37 °C for different time intervals. The infected cells drawn at 1, 2 and 3 h of infection were washed in 1 × PBS and lysed by adding 200 μL of 0.5% Triton X-100 and 800 μL of 1 × PBS to release internalized bacteria. All the experiments were repeated three times. Internalized bacteria were enumerated by spread plating ten-fold serial dilutions on LB agar plates.

### 2.8. Cytotoxicity Assay

Infection of the HeLa cell was carried out as described earlier. One mL of the culture supernatant was collected 0.5, 1, and 2 h postinfection to estimate the percentage of cytotoxicity. The ability of *V. vulnificus* to induce cytotoxicity in HeLa cells was studied using a Cytoscan-LDH cytotoxicity assay (G-Biosciences, St. Louis, MI, USA). The amount of cytosolic enzyme, lactate dehydrogenase (LDH), which is released upon cell lysis, was quantitated.

### 2.9. RNA Extraction and DNase Treatment 

The infection of HeLa cells lines was performed as mentioned earlier. At 15, 30, 45 and 60 min of incubation, cells were detached using trypsin-EDTA solution. RNA extracted at 0 min served as control. RNA extraction was done using the TRIzol method (Takara, Kyoto, Japan). DNase treatment was carried out as per the manufacturer’s protocol (Thermo Fisher Scientific Inc, Bartlesville, OK, USA) for the removal of genomic DNA contaminant from the preparations. The concentration of RNA was normalized to 100 ng/µl and kept at −80 °C until use.

### 2.10. qPCR Assay

The real-time PCR (qPCR) assay was done using iTaq Universal SYBR Green one-step kit (Bio-Rad, Hercules, CA, USA). Primers for the PCR assays were designed taking the *V. vulnificus* reference sequence from NCBI (accession number NZ_CP037931.1 and CP037932.1). Details of primers used in this study are provided in Table S6. Primer concentration was optimized for all the genes and used in the subsequent experiments. Serially diluted cDNA was used to analyze the amplification efficiency of primers. Melt curve analysis was performed to rule out random amplification. mRNA transcripts were quantified using the 2^−ΔΔct^ formula [45]. The *rpoA* gene was used as an internal control. The qPCR was carried out in 10 µL reaction volume consisting 5 µL of 2 × universal SYBR green master mix, 200 nM of forward and reverse primers, 0.125 µL of reverse transcriptase, 1 µL of template RNA (100 ng/uL) and molecular-grade water to adjust the volume. The PCR was performed in a CFX96 Touch Real-time PCR detection system, (Bio-Rad, Hercules, CA, USA) with following thermocycling conditions—cDNA synthesis at 50 °C for 10 min, initial denaturation at 95 °C for 3 min followed by 40 cycles of denaturation at 95 °C for 10 s, primer annealing at 55 °C for 20 s and final extension and elongation at 72 °C for 20 s. 

### 2.11. Evaluation of Cell Structural Damage by Immunofluorescence Assay 

HeLa cells were grown to confluency on a surface-treated coverslip placed in a sterile 24-well tissue culture plate wherein DMEM supplemented with 5% fetal bovine serum was added. Media was removed from the wells and cells were washed thrice with 1 × PBS to remove the residual antibiotics and this was followed by infection. Bacterial cells were grown overnight in LB at 37 °C and used to infect HeLa cells at an MOI of 1:5. Following infection, the Hela cells were incubated at 37 °C for 15 min, 30 min and 1 h. Media was removed from the well and the coverslip with adherent cells was washed thrice with 1 × PBS and then fixed in 3.7% formaldehyde for 20 min at room temperature (28 °C) on a rocker. This was followed by washing with 1× PBS thrice for 5 min and staining with Phalloidin-iFlour 488 Conjugate (AAT Bioquest, Sunnyvale, CA, USA) (1×) in 1 × PBS (1% BSA) for 2 h at room temperature (28 °C) in a rocker. After 2 h, phalloidin solution was removed and cells were washed thrice with 1 × PBS. Nuclear staining was performed by incubating the cells with 300 nM 4′,6-diamidino-2-phenylindole (DAPI) for 30 min at room temperature in a rocker. Cells were washed thrice in 1 × PBS and the coverslip mounted on the slide using flouromount (Sigma-Aldrich, St. Louis, MI, USA). Images were captured using a fluorescence microscope (Olympus-BX53).

### 2.12. Statistical Analysis

For statistical analysis of data, Factorial ANOVA was performed with the Bonferroni using SPSS 16.0 software (SPSS Inc., Chicago, IL, USA). A value of *p* < 0.05 was considered significant. 

## 3. Results

### 3.1. Isolation and Phenotypic Characterization of V. vulnificus from Seafood

This study was conducted to understand the genetic diversity, virulence attributes and pathogenic potential of *V. vulnificus* isolated from seafood harvested along the coast of Mangaluru, India. Of the 70 seafood samples examined (clams, oyster, mussel, crab, shrimp, sardine, mackerel and marine sediment), 21 (30%) samples were positive for *V. vulnificus* among which 90.47% of positive samples were clams and oyster. Isolates obtained that were opaque showed great phenotypic similarity. All the isolates tested that were positive for indole reaction, D-mannitol fermentation and decarboxylation of lysine belonged to biotype 1. [46]. Most of the isolates were negative for decarboxylation of ornithine and lactose fermentation which is largely unusual for this organism. All the isolates showed hydrolysis of starch, gelatin, casein and produced hemolysins against human erythrocytes. The details of the biochemical and physiological characteristics of *V. vulnificus* examined in this study are summarized in Table S1.

### 3.2. Genotyping of V. vulnificus Isolates

**Figure 1:**
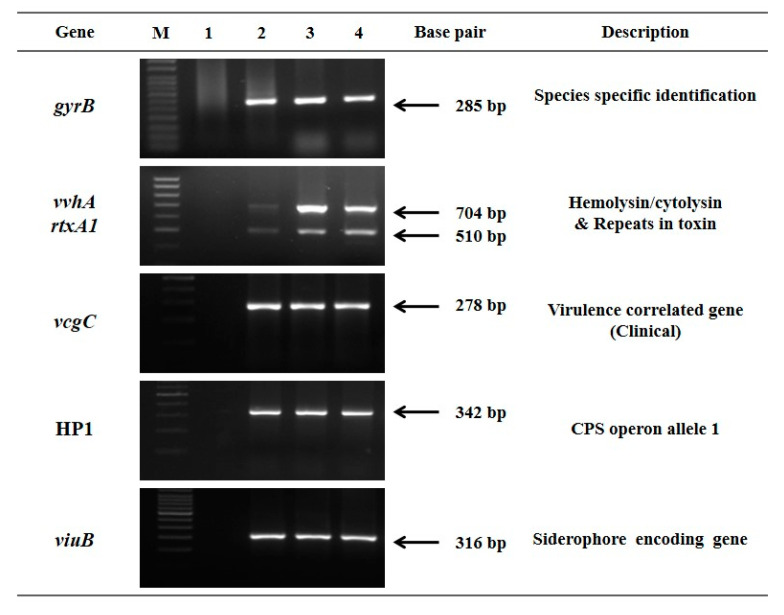
Agarose gel electrophoresis image showing PCR amplification of genes tested for *V. vulnificus* isolates. Lane M: Marker, Lane 1: Negative control, Lane 2: Positive control, Lane 3 and 4: Representative seafood isolates of *V. vulnificus.* Gel image was acquired using Gel Doc XR System and controlled by Image Lab Software (Bio-Rad, Hercules, CA, USA). A 50 bp DNA marker was used for *gyrB* gel electrophoresis and a 100 bp marker was used during electrophoresis of remaining amplicons.

All biochemically suspect isolates of *V. vulnificus* were positive for *gyrB* (species-specific marker) confirming the isolates as *V. vulnificus* [7]. Further, all the isolates possessed virulence-associated genes coding for the protein hemolysin/cytolysin (VvhA), MARTX and siderophore gene (*viuB*) (Figure 1). 

**Figure 2:**
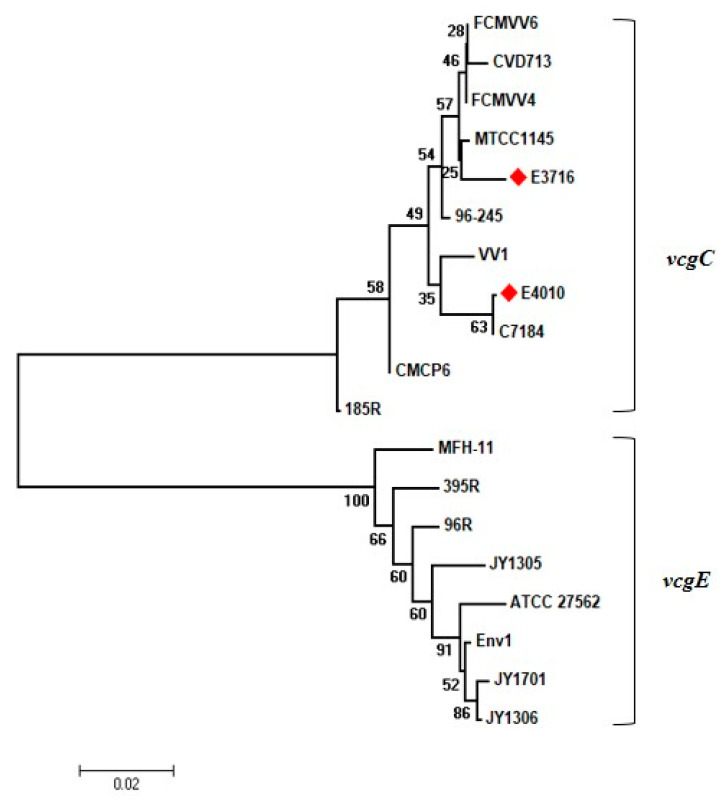
Phylogenetic analysis of the *vcg* alleles of *V. vulnificus*. Phylogeny was estimated by the Neighbor-joining method (*p*-distance) using the sequence information obtained from the NCBI. Numbers in the phylogenetic tree are the representation of percentages of bootstrap support from 1000 replicates. E3716 and E4010 are representative seafood isolates obtained in this study.

Nucleotide polymorphisms were checked within different genetic loci such as the *vcg* allele, and CPS allele as per the previously published protocol, to analyse the association between isolation source and genotypic characteristics [13,14]. Interestingly, all the isolates exhibited exceptional similarity to the genetic characteristics commonly observed in clinical isolates. In the case of *vcg* allele typing, all the isolates were amplified only by P1 and P3 primers. The analysis of the partial *vcgC* gene sequence of the representative isolate (GenBank accession number MK814530.1 and MK814529.1) showed that tested isolates clustered along with clinical isolates (Figure 2). Variation within the CPS operon strongly correlates with two distinct genotypes, HP1, which is commonly associated with the clinical genotype, and HP2 with the nonclinical type. To examine the distribution of the CPS allele among isolates, allele-specific PCR was performed. All the isolates had CPS allele 1 which corresponds to the clinical genotype. Detailed information of the genotyping is presented in Table S2. 

### 3.3. Serum Resistance/Sensitivity Assay 

**Figure 3:**
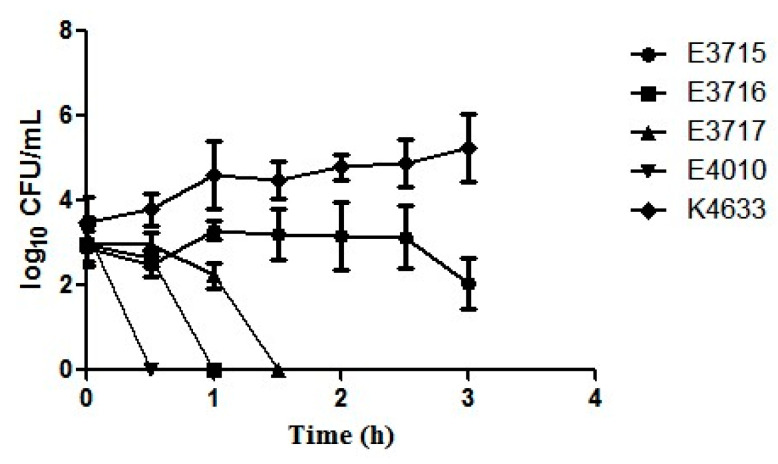
Survival of C-type environmental strains of *V. vulnificus*. E3715 (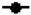), E3716 (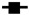), E3717 (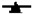), E4010 (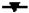), and a clinical strain K4633 (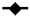) exposed to serum for 3 h. Error bars represent the standard error of the mean of three replicates each.

Four environmental and one clinical strain were examined for their ability to overcome the inhibitory effects and survival in undiluted serum. A significant difference was seen in the survivability between seafood and clinical isolates (*p* < 0.001). The clinical isolate successfully resisted the bactericidal activity of serum showing a 2 log increase at the end of 3 h. The seafood isolates E4010, E3716, E3717 lost their viability within 0.5, 1 and 1.5 h, respectively, whereas E3715 survived until 3 h, but with a 1 log reduction (Figure 3). Additionally, isolate E4010 was checked for the ability to survive in different dilutions of serum. A total of 6.25% and 12.5% of serum was resisted by the bacteria, whereas 25% and 50% serum proved lethal, with the cells losing their viability within 1 and 0.5 h, respectively (data not shown). Inactivated serum did not inhibit the survival and replication of the bacteria (data not shown). 

### 3.4. Adherence Ability of Environmental Strains of V. vulnificus

Adherence to the host cell is the initial step of the invasive process to gain entry into the bloodstream. Adherence assays were performed in cultured HeLa cells and *V. vulnificus* enumerated at 0.5, 1 and 2 h postinfection. There was approximately a 1–2 log increase in the bacterial counts at the end of 2 h. Adherence capacity ranged from 8–17%, 11–23% and 50–100% at 0.5, 1 and 2 h, respectively. The number of bacteria per HeLa cell ranged from 4–39 at the end of 2 h and was negligible during the initial hours of postinfection (Table 1). Comparison of the environmental isolates with the clinical isolate did not show any significant difference (*p* > 0.05). Gentamicin protection assay was performed to enumerate the number of intracellular bacteria at the time of infection. Due to rapid disintegration of actin (discussed under immunofluorescence assay below), we could not reveal any intracellular bacteria.

### 3.5. Cytotoxic Effect of V. vulnificus on HeLa Cells 

**Figure 4:**
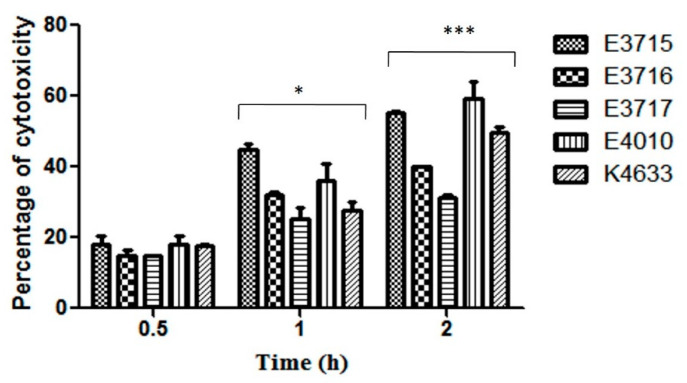
HeLa cells were infected with *V. vulnificus* at a multiplicity of infection (MOI) of 5. Percentage of cytotoxicity was measured at 0.5, 1, and 2 h of postinfection by LDH release assay. The data represents means and standard errors from three independent experiments. Factorial ANOVA was performed to analyze the difference between the strains (* *p* < 0.05 and *** *p* < 0.001).

All the isolates were checked for their cytotoxicity to HeLa cells by LDH assay. The percentage cytotoxicity at 0.5, 1 and 2 h postinfection was recorded. Nearly 20% of cytotoxicity was observed within 0.5 h postinfection with no difference among the isolates (*p* = 0.053). However, cytotoxicity varied at 1 and 2 h postinfection (*p* < 0.05). The highest cytotoxicity recorded was 44.95% at 1 h and 59.27% at 2 h postinfection by environmental strain E3715 and E4010, respectively (Figure 4). Microscopic examination of HeLa cells infected with *V. vulnificus* showed cell rounding and disruption. Uninfected HeLa cells had a uniform monolayer and normal cell appearance throughout the experimental period and served as control.

### 3.6. Expression of Virulence Genes

**Figure 5:**
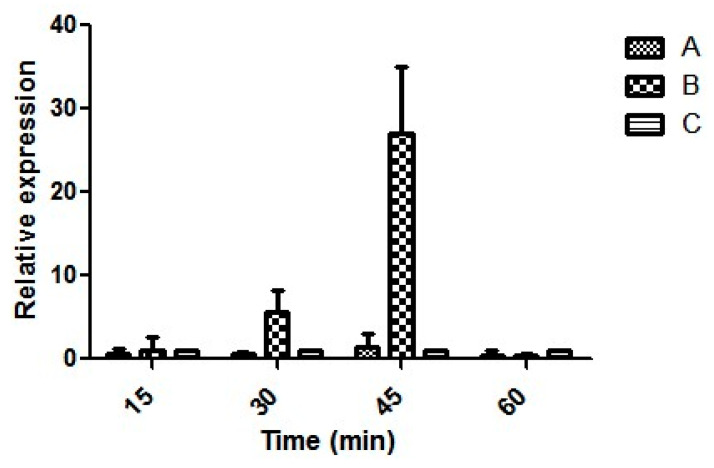
Relative expression of the *rtxA1* gene in seafood isolate E4010 (**A**) *V. vulnificus* grown in Luria Bertani (LB) broth overnight at 37 °C incubated in Dulbecco’s Modified Eagle’s Medium (DMEM), (**B**) *V. vulnificus* grown in LB broth overnight at 37 °C was used to infect to HeLa cells at an MOI of 1:5, and (**C**) HeLa cells harvested at 0 h postinfection (control).

All the isolates obtained were similar in terms of genotypic and phenotypic attributes. Hence, the expression of virulence genes was performed using one representative isolate E4010. Moreover, the same isolate was studied in detail for its acid stress response in our previous study [47]. The relative expression ratio of each transcript tested remained at the baseline, whereas expression of *rtxA1* increased after 30 min postinfection and remained high until 45 min postinfection. There was a 7- and 23- fold increase in the expression of *rtxA1* at 30 and 45 min postinfection, respectively (Figure 5). *pilA* did not show any expression, whereas *vvhA*, *vvpE*, *ompU* and *flp* were constituently expressed (data not shown) both during the infection of HeLa cell and in the media. *rtxA1* showed contact-mediated increased expression and remained low in the media without the cells.

### 3.7. Effect of V. vulnificus Infection on HeLa Cell Morphology and Actin Perturbation

**Figure 6:**
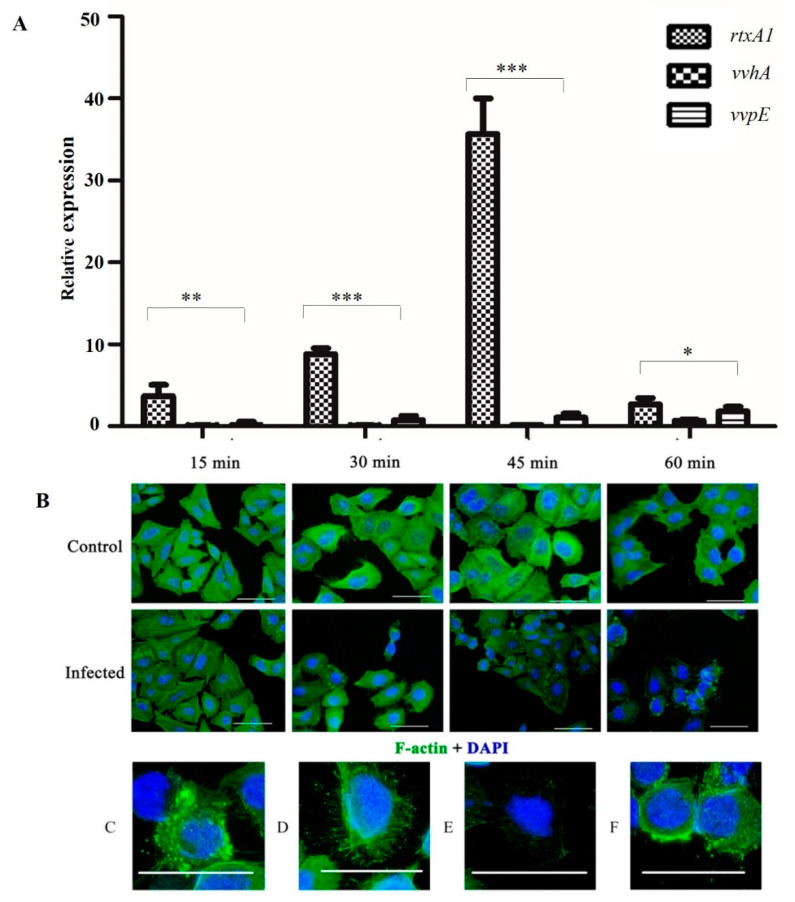
Effect of adhering *V. vulnificus* on F-actin network and nucleus of HeLa cells at different time points. HeLa cell monolayers were infected at an MOI of 5 and incubated for 1 h at 37 °C. (**A**) The relative expression level of *rtxA1*, *vvhA* and *vvpE* at the time of infection. Results are mean ± SD of triplicate experiments. Asterisks indicate significant differences in the expression of genes (* *p* < 0.05, ** *p* < 0.01 and *** *p* < 0.001). (**B**) Immunofluorescence images of HeLa under 20×. Scale bar corresponds to 50 µM. Images are overlays of actin (green) and nucleus (blue) signals. Descriptive features of HeLa cell at 1 h of postinfection; blebbing (**C**), discrete actin bundles (**D**), diffuse actin bundles (**E**), cell rounding (**F**).

To investigate the effect of *V. vulnificus* E4010 infection on HeLa cells, an immunofluorescence assay was performed using phalloidin and DAPI staining. The extent of cellular damage was determined by observing perturbation in F-actin assembly as a function of time, postinfection. Contrasting variation in F-actin organization was evident in infected cells compared to control. Phalloidin staining showed a polarized dense network of actin filaments in control cells whereas compact actin integrity was compromised in infected cells. Actin fiber disintegration started 30 min postinfection and almost complete disintegration was observed at 1 h (Figure 6). However, no nuclear changes were observed in response to *V. vulnificus* infection. Results show that infection with *V. vulnificus* causes progressive disorganization of F-actin assemblages.

## 4. Discussion

*V. vulnificus,* being an autochthonous organism, is routinely isolated from seafood and marine environments in India. The incidence in marine fish ranges from 13 to 16%. In molluscan shellfish such as clams, it is 38.5%, while in oysters, 43% to 75% has been reported [5,6,7,38]. We isolated *V. vulnificus* in oyster and clams with a higher prevalence rate of 44%. A pertinent research question in this study relates to the virulence of *V. vulnificus* strains associated with seafood in India, considering that there are very few reports on *V. vulnificus* infection. [9,10,36]. This study presents evidence of the virulence attributes among *V. vulnificus* isolated from tropical seafood.

*V. vulnificus* is heterogeneous and includes both virulent and avirulent strains. Several attempts to differentiate the isolates as pathogenic and nonpathogenic based on phenotypic and genotypic characteristics have been made. Biochemical characteristics and mouse virulence assays did not show any significant difference between clinical and environmental isolates [16,19,48]. All the tested isolates in the present study possessed *vvhA*, demonstrating hemolysis of human erythrocytes. *viuB,* a siderophore-encoding gene, was present in all the tested isolates and they concurrently exhibited siderophore activity in CAS agar. In addition, all the isolates possessed *rtxA1* coding for a repeat toxin, which is considered an important virulence attribute. Based on polymorphism in CPS operon and the *vcg* gene, it was confirmed that seafood isolates in the present study are of the clinical type. Environmental isolates possessing the C genotype have been previously reported from this region [49].

Gastric acidity is one of the initial barriers experienced by the bacteria present in food, and pathogens have to overcome this host factor to successfully colonize and infect. In our previous study, we have demonstrated that seafood isolates were able to generate adaptive acid tolerance response and have a functional lysine decarboxylation system to successfully transit across the gastric barrier [47]. Bacterial adherence to the host cell surface and invasion through the gastrointestinal barrier is a crucial factor to determine its potential to cause systemic infection. Isolates tested in this study showed a time-dependent increase in the adherence to the HeLa cells comparable with the clinical strain, with no discernible difference among isolates. The cytotoxicity experiment showed that *V. vulnificus* induces cell death in a time-dependent manner, which is in agreement with a previous study [50]. RtxA1 is the important virulence factor of *V. vulnificus,* responsible for cytotoxic and cytopathic changes, including necrosis and apoptosis during the time of infection [51,52]. To correlate the cytotoxicity and actin disintegration caused by seafood isolates of *V. vulnificus,* the transcriptional activity of important virulence determinant genes was studied. Actin depolymerisation was observed within 1 h postinfection wherein *rtxA1* expression was dominant, which correlated well with cytotoxicity. This also explains why we could not detect any intracellular bacteria, which is most likely due to the rapid disintegration of F-actin mediated by *rtxA1* upon infection [53]. Kim et al. [50] reported contact-mediated *rtxA1* expression in clinical strains and this study demonstrated this phenomenon in the seafood isolate tested. Nevertheless, all the other core putative virulence genes such as *vvhA* and *vvpE ompU* and *flp* were constitutively expressed. *pilA* failed to amplify, which might be due to the highly divergent *pilA* in different isolates of *V. vulnificus* or lack of its expression [54]. Integrating all the observations from gene detection to the expression analysis, seafood isolates tested in this study demonstrated toxin-mediated cytotoxicity, while further studies are required to validate the results obtained.

Toxins facilitate entry into the blood stream, but survival thereafter depends on the bacteria’s ability to fight against the immune components of blood. Complement-protein mediated killing is part of the innate immune mechanism to clear the pathogens from blood [3]. It has been noted that *V. vulnificus* with opaque colony morphologies have better resistance towards antimicrobial effects of serum complement proteins and show significant survival in serum [33,55]. Seafood isolates analysed in the present study showed high or moderate serum sensitivity, whereas the clinical strain successfully resisted the bactericidal activity of serum. Isolate E3715 showed higher survival ability compared to other seafood isolates. This suggest that seafood isolates have different survival potential in serum. Factors governing this difference need to be studied. All the seafood isolates were opaque in terms of colony morphology and showed serum sensitivity, suggesting a lack of association between opaque colonies and serum resistance. Overall findings highlight the need to further investigate the genetic factors that govern disparity in the serum survival of seafood-associated environmental isolates and those derived from clinical sources.

## 5. Conclusions

Determining the factors responsible for the virulence of *V. vulnificus* has been challenging. Identifying the virulent strains and controlling them at its source is the need of the day. The isolates tested possessed the *vcgC* gene sequence with high similarity to that in the clinical strain. Overall results suggest that the seafood isolates tested in this study possess a functional RtxA1 which could help in initiating the infection. Nevertheless, other putative virulence genes such as *vvpE* encoding an extracellular protease*, vvhA* encoding hemolysin*, flp* encoding tad pilin and *ompU* encoding fibronectin-binding protein were also constitutively expressed. However, despite belonging to the clinical genotype and presenting an opaque colony characteristic, the isolates from seafood, when tested for survival in serum, were inhibited as compared to the clinical strain indicating the ability of the clinical strain to survive in blood and cause systemic disease (*p* < 0.001). From the point of virulence, many putative virulence attributes such as toxins and other cell components have been suggested for the lethality caused by the bacteria, but the precise mechanism of action of many of these remains to be elucidated. It is increasingly clear that hitherto described genotypic attributes are not sufficient to predict the virulence of *V. vulnificus*. Much remains to be studied on the pathogenicity attributes of the clinical and environmental isolates to make a distinction.

## Figures and Tables

**Table 1:** Adherence ability among different strains of *V. vulnificus.*

Strain	Percentage of Adherence		
	0.5 h	1 h	2 h
E3715	8 ± 1.5	11.3 ± 3.05	>100
E3716	10.1 ± 2.1	12 ± 2.5	>100
E3717	6.5 ± 1.6	17 ± 1.4	50–100
E4010	17 ± 2.8	16.5 ± 3.5	>100
K4633	17.3 ± 3.05	23.6 ± 4.8	>100

## Supplementary Materials

The following are available online at https://www.mdpi.com/2076-2607/8/7/999/s1, Table S1: Biochemical and physiological characteristics of *V. vulnificus*, Table S2: Genotypic attributes of *V. vulnificus* analysed in this study, Table S3: Details of isolation of *V. vulnificus* from seafood, Table S4: Details of sampling location and coordinates, Table S5: Details of primers used for species specific confirmation, genotyping and virulence gene detection, Table S6: Details of primers used for real time PCR.
