# Supplementary material for: Exploring the Pathogenic Potential of Vibrio vulnificus Isolated from Seafood Harvested along the Mangaluru Coast, India
Source: Microorganisms. 2020 Jul 4;8(7):999. doi: 10.3390/microorganisms8070999 (PMC7409051; doi:10.3390/microorganisms8070999)
Supplement: Supplementary file 1 [file microorganisms-08-00999-s001.pdf]

Supplementary material

Exploring the Pathogenic Potential of *Vibrio vulnificus* Isolated from Seafood Harvested Along the Mangaluru Coast, India

Caroline D'Souza, Kattapuni Suresh Prithvisagar, Vijay Kumar Deekshit, Indrani Karunasagar, Iddya Karunasagar, Ballamoole Krishna Kumar*

Nitte University Centre for Science Education and Research, Nitte (Deemed to be University), Deralakatte, Mangaluru-575018, Karnataka, India.

Table S1: Biochemical and physiological characteristics of *V. vulnificus*

Biochemical trait	Result (n=21)
Indole test	+
Salt tolerance 0%	-
Salt tolerance 3%	+
Salt tolerance 8%	-
Salt tolerance 11%	-
Citrate test	+
Arginine decaboxylase	-
Lysine decaboxylase	+
Ornithine decaboxylase	Variable
Mannitol fermentation	+
Lactose fermentation	Variable
Oxidase test	+
Catalase test	-
O/F	+/+
Lecithinase activity	-
Starch hydrolysis	+
Caseinase activity	+
Hemolysis (human erythrocyte)	+
Gelatinase activity	+
Siderophore	+

Table S2: Genotypic attributes of *Vibrio vulnificus* analysed in this study

Genotypic attributes	Result (n=21)
viuB (vulnibactin siderophore)	+
vcgC (Virulence correlated gene, Clinical type)	+
vcgE (Virulence correlated gene, Environmental type)	-
HP1 (CPS Operon allele 1)	+
HP2 (CPS Operon allele 2)	-
vvhA (*Vibrio vulnificus* hemolysin A)	+
rtxA1 (Repeats in toxin)	+

Table S3: Details of isolation of *V. vulnificus* from seafood

Type of sample	Total no of sample	No of samples positive for *V. vulnificus*
Bivlaves (clams, oyster & mussel)	44	19
Crustaceans (crab, shrimp)	16	2
Fin fishes (sardine, mackerel, sole fish)	6	-
Marine sediment	4	-
**Total**	**70**	**21**

Table S4: Details of sampling location and coordinates

Sampling station	Coordinates
Mulki	13°5’18.6936” N<br>74°46’57.396” E
Mangalore old port	12°51’37.2024” N<br>74°49’54.9336” E
Sasihithlu	13°3’58.1724” N<br>74°46’47.5176” E
Ullala	12°49’53.4972” N<br>74°50’14.0208” E
Manjeshwara	12°42’21.1896” N<br>74°53’16.4832” E
Bengre	12°52’58.5192” N<br>74°49’5.0808” E

Table S5: Details of primers used for species specific confirmation, genotyping and virulence gene detection

Primer code	Primer sequence (5'-3')	Product size (bp)	Annealing temperature (°C)	Reference
gyrB	(F): GTCCGAAGTGGAATCCTTCA<br>(R): TGGTTCTTACGGTTACGGCC	285	63	Kumar et al. 2006
vvhA	(F): GACTATCGCATCAACAACCG<br>(R): AGGTAGCGAGTATTACTGCC	704	55	Lee et al. 1998
rtxA1	(F): TGAAGGTGTCGTGGTTACA<br>(R): GTCAGGTCCTTCTCGACAGC	510	60	Acharya et al. 2013
viuB	(F): GGTTGGGCACTAAAGGCAGAT<br>(R): TCGCTTTCTCCGGGGCGG	316	60	Panicker et al. 2004
vcgC	(F): AGCTGCCGATAGCGATCT<br>(R): CGCTTAGGATGATCGGTG	278	55	Rosche et al., 2005
vcgE	(F): CTCAATTGACAATGATCT<br>(R): CGCTTAGGATGATCGGTG	278	55	
HP1	(F): TTTGGGTTTGAAAGGCTTG<br>(R): GTGCCTTTGCGAATTTTGAT	342	50	Han et al. 2009
HP2	(F): TTCCATCAAACATCGCAGAA<br>(R): CTTTTGTCCGGCTTCTATGC	152	50	

Table S6: Details of primers used for real time PCR

Primer code	5'-3'	Product size (bp)	Reference
rtxA1	CCGCATGGTCAAGTAAGGTT	175	This study
	TGTCAGGCAATGAGAAGCAC		
vvhA	CTATCGTGCACGCTTTGGTA	213	This study
	ACCGTTTTGTCACCGTTCTC		
vvpE	AGCATGGAGCAAAGAAGCAT	183	This study
	CCTTGCAATGCTCTGTCGTA		
Flp	TGACAAAAGCGATTGAGTGG	114	This study
	CAGACATAGCAACGGCAATAATC		
ompU	ACACCGTATAGGCCGTCTTG	166	This study
	CTGGTGTTAACGCTGCTGAA		
pilA	GAGTTAATGATAGTGGTGGCG	225	This study
	CAGTGTTATGGTACCAAGAGC		
